# Comparative analyses of fecal microbiota in Tibetan and Chinese Han living at low or high altitude by barcoded 454 pyrosequencing

**DOI:** 10.1038/srep14682

**Published:** 2015-10-07

**Authors:** Long Li, Xin Zhao

**Affiliations:** 1 College of Animal Science and Technology, Northwest A&F University, Yangling, Shaanxi, People’s Republic of China; 2 College of Animal Science, Agricultural and Animal Husbandry College of Tibet University, Nyingchi, Tibet, People’s Republic of China; 3 Department of Animal Science, McGill University, 21,111 Lakeshore, Ste. Anne de Bellevue, Quebec, Canada, H9X 3V9

## Abstract

Knowledge about the impact of altitude and ethnicity on human gut microbiota is currently limited. In this study, fecal microbiota from 12 Tibetans (T group), 11 Chinese Han living in Tibet (HH group) and 12 Chinese Han living in Shaanxi province (LH group) were profiled by 454 pyrosequencing. Analysis of UniFrac principal coordinates showed significant structural changes in fecal microbiota among the three groups. There were significant differences in the composition of fecal microbiota among the three groups at phylum and genus levels. At the phylum level, the fecal samples of HH and T groups had higher relative abundances of *Firmicutes*, whereas the LH group had a higher relative abundance of *Bacteroidetes*. These changes at the phylum level reflected different dominant genus compositions. Compared with the LH group, changes of *Firmicutes* and *Bacteroidetes* were mainly due to a significant decrease of *Prevotella* in the HH group and were primarily attributable to significant decreases of *Bacteroides* and *Prevotella* as well as a significant increase of *Catenibacterium* in the T group. In conclusion, our results suggest that high altitude may contribute to shaping human gut microbiota. Genetic and dietary factors may also explain the different microbiota compositions between Tibetan and Chinese Han.

The microbes in our bodies collectively make up to 100 trillion cells and they encode 100-fold more unique genes than our own genome^1^. The abundant and diverse microbiota in the gastrointestinal tract plays an important role in host physiology, including immunomodulation, maintenance of the gut barrier and involvement in nutrient metabolism^2^. The functions of microbiota in the gastrointestinal tract are largely determined by the composition.

Different geographic origins of humans may result in diverse compositions of gut microbiota, due to distinctive life environments, genetic background or dietary habits. De Filippo *et al*. (2010)^3^ found that children living in rural Burkina Faso had a higher bacterial diversity and a lower Firmicutes/Bacteroidetes (F/B) ratio than those of Italian children. In addition, genera *Xylanibacter* and *Prevotella*, which are related to cellulose and xylan hydrolysis, were exclusively present in gut microbiota of Burkina Faso children^3^. Similar research performed by Lin *et al*. (2013)^4^ revealed that children living in Bangladesh showed a higher bacterial diversity and different community composition as compared to children living in the United States of America. An across-country survey performed by Yatsunenko *et al*. (2012)^5^ also showed that there were significant differences in the phylogenetic composition of fecal microbiota among individuals living in three different countries (Venezuela, Malawi and United States of America) and the authors attributed the differences to different geographic origins and age. In contrast, Arumugam *et al*. (2011)^6^ found no significant structural changes of gut microbiota of subjects from 6 different countries and no effects of host properties such as body mass index, age, or gender on gut microbiota structure. The conflicting results from various studies highlight the necessity that a wider range of human living in different areas should be studied to characterize the global extent of human-associated microbial diversity^4^,^7^,^8^ and reflect the compounding effects of different life environment, genetic background and dietary habit. The dietary factor has been recognized to have a dominant role in different gut microbiota^3^. As for genetic background, Khachatryan *et al*. (2008)^9^ was the first to show that a gene (MEFV) mutation could affect gut microbiota in humans. Subsequently, Goodrich *et al*. (2014)^10^ compared microbiota in more than 1,000 fecal samples obtained from the TwinsUK population and identified many microbial taxa whose abundances were influenced by host genetics. In addition, environmental change also results in altered gut microbiota. For example, studies have shown that short exposure to high-altitude can alter the composition of gut microbiota^11^,^12^.

Tibet is a typical high altitude area in China and has special environment conditions, such as low barometric pressure, low temperature, low humidity and high radiation. The population in Tibet is mainly composed of Tibetans and Chinese Han. Indigenous Tibetans have been living at high altitude for many years. The hypoxia of altitude (hypobaric hypoxia) would thus have exerted substantial evolutionary selection pressure. Tibetans adapt well to the hypoxia environment because of their different genotypes formed by long time natural selection, called genetic adaptation^13^. Recently, genetic studies conducted on the hypoxia adaptation of Tibetans have found differences in several genes compared with lowland Han^14^,^15^,^16^. For example, hypoxia-related genes EPAS1 and EGLN1 were suggested to be responsible for genetic adaptation of high-altitude hypoxia in Tibetan^14^,^15^,^16^,^17^. However, whether similar genetic changes have occurred to affect gut microbiota is not clear. Tibetans also have different diet habits, culture and lifestyles from Chinese Han^18^,^19^. By using quantitative polymerase chain reaction (qPCR), Kowk *et al*. (2014)^8^ compared 10 major bacterial groups from the 4 dominant phyla of the common core gut microbiota between Chinese Han and Tibetans and found different fecal bacterial composition and a weak difference in structure. However, due to technical limitations, qPCR used by Kowk *et al*. (2014)^8^ is not suitable to study comprehensive changes of microbiota. Therefore, one objective of this study was to compare different ethnic origins (Han and Tibetan) on gut microbiota by 454 bar-code pyrosequencing.

Individuals from low-altitude populations (Han) suffer from a number of potential diseases specifically related to the low levels of oxygen, when they move to live at high altitude^20^. Unlike Tibetan, Chinese Han who recently migrated and are living in Tibet may adapt to the hypoxia environment in different ways. A series of physiological changes may take place such as ventilation, cardiac function, oxygen delivery, muscle structure, oxygen consumption, and metabolism^21^,^22^. Short exposure to high-altitude could affect the composition of gut microbiota^11^,^12^. However, whether the physiological changes in the Chinese Han’s acclimatization to high altitude are accompanied by changes of gut microbiota is still unknown. So another objective of this study was to investigate the effect of altitude on gut microbiota by comparing Chinese Han living at low altitude to those living at high altitude.

## Results

### Characteristics of pyrosequencing results

**Figure 1:**
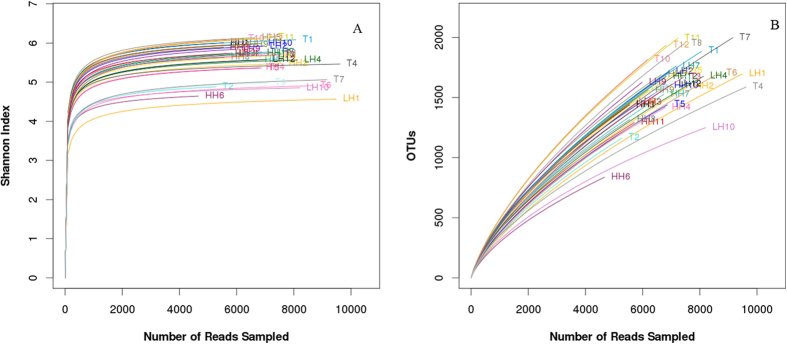
Rarefaction curves and Shannon-Wiener curves of each sample. Shannon-Wiener curves (**A**) and rarefaction curves (**B**) were all calculated at the 97% similarity level with pyrosequencing data in microbiota from groups of LH, HH and T. LH = Chinese Han living in Shaanxi; HH = Chinese Han living in Tibet; T = Tibetans.

In total, 442,737 raw reads were obtained from all 35 fecal samples. After filtering, 294,698 high-quality sequences were produced, with an average of 6923 ± 1191 reads per sample. The total number of OTUs at the 97% similarity level was 22,913 (Table 1 and Table S1). The Shannon-Wiener curve of all samples already reached a plateau at this sequencing depth (Fig. 1), suggesting that the sequencing was deep enough. Good’s coverage estimations revealed that 80.3% to 91.0% of the species were obtained in all of the samples. Estimators of the community richness (Chao) and the diversity (Shannon index) are summarized in Table 1. There were no statistically significant differences in the community richness estimator (Chao) and the diversity estimator (Shannon index) among three groups (Table 1).

### Taxonomy-based comparisons of fecal microbiota at the phylum and genus levels among three groups

**Figure 2:**
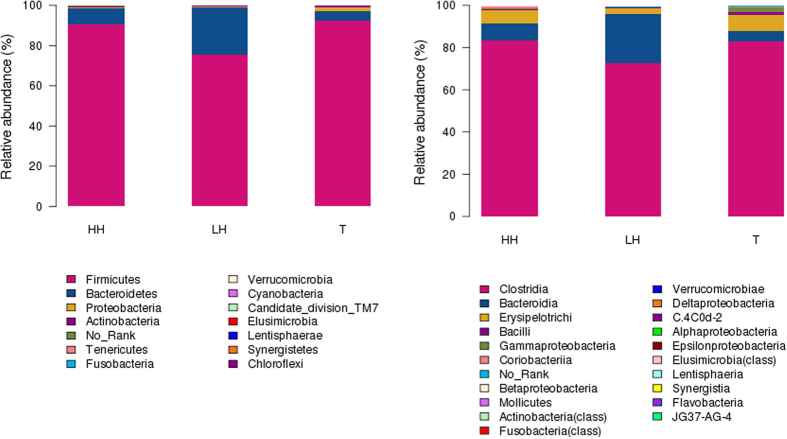
Relative abundance of bacterial phyla (A) and classes (B) in fecal microbiota of three groups. LH = Chinese Han living at the lowland; HH = Chinese Han living in Tibet; T = Tibetans.

Overall microbiota compositions for each group at the phylum and class levels are shown in Fig. 2. There were 14 phyla and 21 classes in the fecal samples. The dominant phyla of all groups were *Firmicutes*, *Bacteroidetes*, and *Proteobacteria* while the dominant classes of all groups were *Clostridia*, *Bacteroidia* and *Erysipelotrichi*. At the phylum level, the most predominant phylum was *Firmicutes*, contributing 75.31%, 90.45% and 92.77% of the fecal microbiota in LH, HH and T groups respectively, followed by *Bacteroidetes*, contributing 23.59%, 7.98% and 4.69% respectively. The microbial composition varied greatly among individuals. Relative abundances of the phyla were analyzed by using a metastats test which either incorporates a paired t-test or uses a paired Wilcoxon rank sum test^23^ and are shown in Table 2. *Firmicutes* was significantly more abundant in the fecal microbiota of HH and T groups than that in the LH group (*p* = 0.022 and 0.006, respectively), while *Bacteroidetes* was significantly less abundant in the fecal microbiota of HH and T groups than that in the LH group (*p* = 0.017 and 0.001, respectively). *Actinobacteria* differed between LH and HH groups (*p* = 0.044). *Cyanobacteria* and *Fusobacteria* exhibited significant differences between HH and T groups (*p* = 0.042 and 0.037, respectively), while *Verrucomicrobia* differed between LH and T groups (*p* = 0.027).

**Figure 3:**
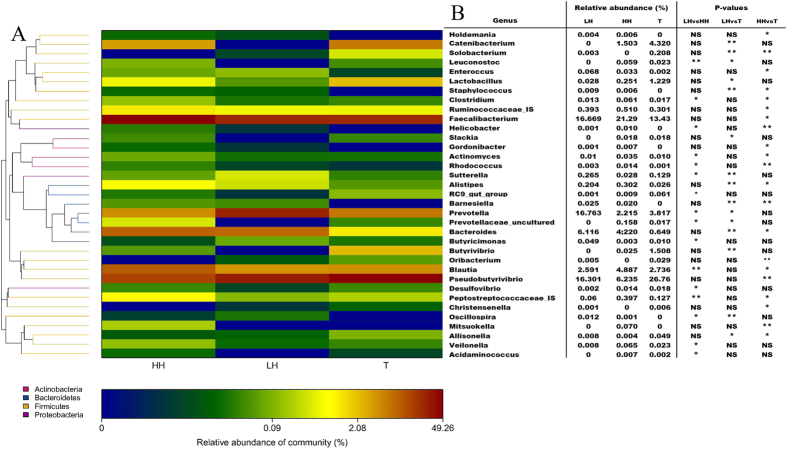
Heatmap of the distribution of the relative abundance of the genera. (**A**) Taxonomic classification of the genera. (**B**) Genera detected and statistical analysis among three groups (Metastats for the sequence count data). LH = Chinese Han living in Shaanxi; HH = Chinese Han living in Tibet; T = Tibetans. Significance: NS > 0.05, *≤0.05; **≤0.01.

Relative abundances of major genera detected and statistical analyses among three groups are shown in Fig. 3. There were 120 genera in fecal samples. At the genus level, the relative abundances of 13 genera were significantly higher in the HH group than in the LH group (*p* < 0.05), including *Acidaminococcus*, *Actinomyces*, *Blautia*, *Butyricimonas*, *Clostridium*, *Desulfovibrio*, *Helicobacter*, *Leuconostoc*, *Peptostreptococcaceae Incertae Sedis*, *Prevotellaceae uncultured*, *Prevotella*, *RC9 gut group* and *Rhodococcus*. On the other hand, 3 genera were significantly lower in the HH group than in the LH group (*p* < 0.05), including *Butyricimonas*, *Oscillospira* and *Sutterella*. The relative abundances of 16 genera were significantly higher in HH group than in T group (*p* < 0.05), including *Actinomyces*, *Alistipes*, *Bacteroides*, *Barnesiella*, *Blautia*, *Clostridium*, *Enterococcus*, *Faecalibacterium*, *Gordonibacter*, *Helicobacter*, *Holdemania*, *Mitsuokella*, *Peptostreptococcaceae Incertae Sedis*, *Rhodococcus*, *Ruminococcaceae Incertae Sedis* and *Staphylococcus*. On the other hand, 5 genera were significantly lower in the HH group than in the T group (*p* < 0.05), including *Allisonella*, *Christensenella*, *Oribacterium*, *Pseudobutyrivibrio* and *Solobacterium*. The relative abundances of 6 genera were significantly higher in the LH group than in the T group (*p* < 0.05), including *Alistipe*, *Bacteroides*, *Barnesiella*, *Prevotella, Staphylococcus* and *Sutterella*. On the other hand, 8 genera were significantly lower in the LH group than in the T group (*p* < 0.05), including *Butyrivibrio*, *Catenibacterium*, *Lactobacillus*, *Leuconostoc*, *Oscillibacter*, *Prevotellaceae uncultured*, *Slackia* and *Solobacterium*.

### Beta diversity of gut microbiota among three groups with multivariate statistics analysis

**Figure 4:**
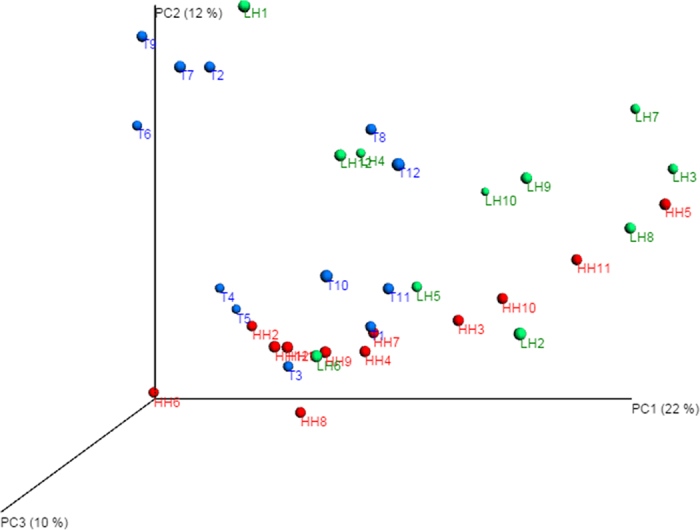
Weighted UniFrac principal coordinates analysis (PCoA) plots based on weighted UniFrac metric. LH = Chinese Han living in Shaanxi (green dots); HH = Chinese Han living in Tibet (red dots); T = Tibetans (blue dots).

A weighted UniFrac principal coordinates analysis (PCoA) was performed to compare the overall structure of gut microbiota of all samples, based on the relative abundance of OTUs (at a 97% similarity level). There was an obvious separation of HH and T groups, while individuals were scattered for the LH group. Pco1, Pco2 and Pco3 accounted for 22%, 12% and 10% of the total variations, respectively (Fig. 4).

Other analysis methods (Jacaard, Bray-curtis and unweighted UniFrac) for the beta-diversity produced similar results (Figure S1).

**Figure 5:**
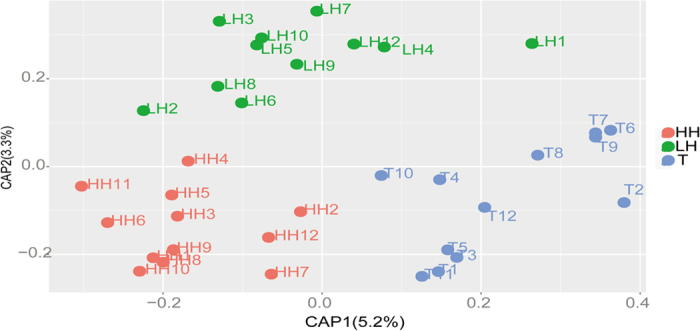
Canonical analysis of principal coordinates (CAP) of human gut microbiota with different ethnicity and altitude. CAP1 was the ethnic axis and CAP2 was the altitude axis. ANOVA test showed significant influence of the two constraints (*p* = 0.001). LH = Chinese Han living in Shaanxi; HH = Chinese Han living in Tibet; T = Tibetans.

Following the PCoA analysis, the statistical analyses showed that microbiota composition was significantly affected by ethnicity with various distance matrixes, but altitude significantly affected the microbiota composition only in weighted UniFrac, unweighted UniFrac and Abund_Jaccard analyses (Table S2). These results are in general agreement with those from the canonical analysis of principle coordinates (CAP) (Fig. 5). However, ethnicity and altitude only accounted for 8.5% of the total variations, indicating that many other factors have effects on the microbiota composition among three groups.

### Real-time qPCR

**Figure 6:**
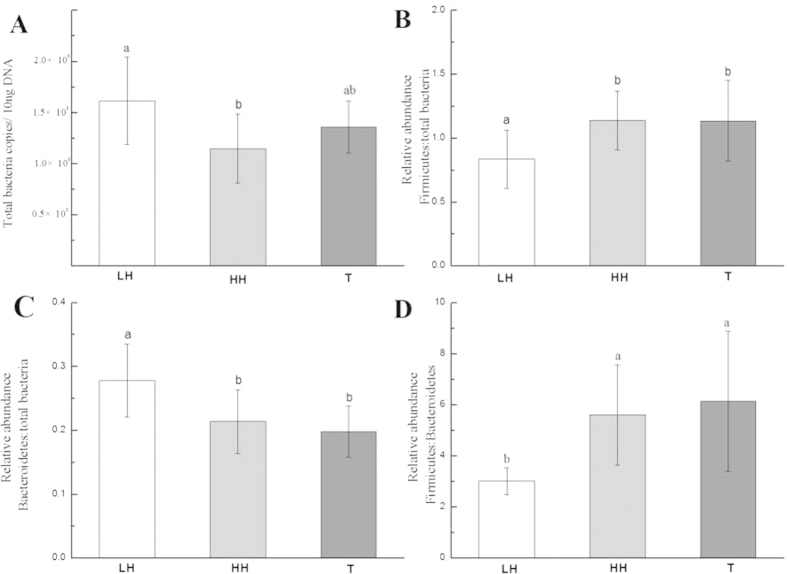
Relative abundance of total bacteria per 10 ng DNA (A), ratios of *Firmicutes*:total bacteria (B), ratios of *Bacteroides*:total bacteria (C), and ratios of *Firmicutes*:*Bacteroides* (D) in the fecal samples of three groups.^a,b^ Different lower-case letters indicate significant differences between groups. LH = Chinese Han living in Shaanxi; HH = Chinese Han living in Tibet; T = Tibetans.

Analysis by real-time qPCR showed total bacterial numbers per 10 ng DNA significantly differed between LH and HH groups (*p* = 0.023). However, total bacterial numbers per 10 ng DNA did not differ between LH and T groups or between HH and T groups (Fig. 6A). Next, the prevalence of the two most abundant phyla of commensal bacteria in the gut, *Bacteroidetes* and *Firmicutes*, were assessed among the three groups. *Firmicutes* dominance was less in the LH group than in the HH and T groups (*p* = 0.013) (Fig. 6B). In contrast to the *Firmicutes* dominance, the *Bacteroidetes* dominance was more in the LH group than in the HH and T groups (*p* = 0.021) (Fig. 6C). Furthermore, the proportional representation of two phyla was significantly shifted in the HH and T groups, in favor of *Firmicutes* (*p* = 0.017) (Fig. 6D).

## Discussion

Our results have provided additional evidence that the geographic origin contributes to shaping human gut microbiota, as previously reported^3^,^4^,^5^. Our study focused on the differences of gut microbiota in human living at high altitude (Tibet) and low altitude (Shaanxi) as well as two ethnic groups (Tibetan and Chinese Han). Study subjects were carefully selected, such that indigenous Tibetans had been living in Tibet for generations, Chinese Han living at high altitude had been living in Tibet more than 4 years and Chinese Han living at low altitude never went to Tibet. Meantime, our participants were within narrow ranges of age (between 18 to 25 years old) and body mass index (BMI) (between 17.8 to 25.1) and all participants were males. Overall, we found different fecal microbiota compositions among the three groups, but no significant differences in the diversity.

The gut microbiota of Tibetans was compositionally distinct from Chinese Han living at high or low altitude in this study. *Firmicutes* and *Bacteroidetes* are two prominent phyla in gut microbiota of humans and have received much attention. Our results showed that Tibetans had a high relative abundance of *Firmicutes* and a low relative abundance of *Bacteroidetes* compared with Chinese Han living at low altitude. However, there was no difference between Tibetans and Chinese Han living at the same high altitude area. These results from pyrosequencing were further verified by qPCR. Interestingly, these changes of the *Firmicutes* and *Bacteroidetes* at high altitude were similar to the shift observed in patients with obesity^24^. However, most of the study subjects in our study had normal BMI according to the standards^25^. In contrast, Kwok *et al*. (2014)^8^ found Chinese Han had a significantly higher F/B ratio than Tibetans. The high ratio of F/B was in response to a high-fat diet and did not affect energy harvesting^26^. A previous large-scale survey revealed the existence of obvious differences in dietary intake between Chinese Han and Tibetans^18^, which was also observed in our study (Table S3). From the questionnaire we found that the frequency for intake of high fat foods such as buttered tea, cheese and meat for Tibetans was higher than that of the Han people. This may explain a high ratio of F/B in Tibetans compared with Chinese Han living in Shaanxi. Nevertheless, the higher ratio of F/B in the HH group than in the LH group could be caused by unknown factors other than high-fat diets, since the two groups of people had similar dietary habits (Table S3).

Compared with the LH group, the changes of the *Firmicutes* and *Bacteroidetes* in the T group were mainly due to significant decreases in genera *Bacteroides* (4.2% vs 0.6%) and *Prevotella* (16.7% vs 3.8%) and a significant increase in genus *Catenibacterium* (0% vs 4.3%). Furet *et al*. (2010)^27^ found that *Bacteroides* and *Prevotella* were negatively correlated with energy intake. Tibetans have higher energy intakes than Chinese Han (13.7 MJ/d vs 11.1 MJ/d)^18^. This may explain low levels of *Bacteroides* and *Prevotella* observed in Tibetans. In addition, Tibetans had a high level of *Pseudobutyrivibrio* (26.8%). The metabolites of genus *Pseudobutyrivibrio* are mainly formate, butyrate and lactate^28^, but the physiological function of *Pseudobutyrivibrio* as a part of the human microbiome is not yet known. While both T and HH groups had a high ratio of F/B, the HH group was mainly due to significant increases in genera *Faecalibacterium* (21.3% vs 13.4%) and *Bacteroides* (6.1% vs 0.6%) and a significant decrease in genus *Pseudobutyrivibrio* (6.2% vs 26.8%) in comparison with the T group. Several genome-wide studies confirmed that several genes showed significantly different genotype frequencies between Tibetans and Chinese Han^14^,^15^,^16^. The gene differences can result in changing the composition of gut microbiota^9^,^10^. Therefore, genetic and dietary factors may explain the differences in the composition of gut microbiota between Tibetans and Chinese Han living in the same high altitude area.

In order to further investigate the environmental factors at high altitude on compositions of gut microbiota, we also compared the gut microbiota between Chinese Han living at low altitude or at high altitude. The total bacteria of Chinese Han in the HH group were significantly decreased in comparison with the LH group. The decreased microbial load could weaken the intestinal ecosystem stability^29^. This result suggests that the intestinal health of immigrants living in Tibet might be easily compromised. The HH group had a higher ratio of F/B than the LH group. These differences were mainly due to a significant decrease in *Prevotella* (2.2% vs 16.7%). *Prevotella* is associated with consumption of a diet rich in carbohydrates^30^. However, Chinese Han living at low or high altitude have similar diet habits and genetic background. Therefore, environmental factors at high altitude are thought to have a certain effect on gut microbiota.

In our study, we found significant differences in genera *Clostridium*, *Desulfovibrio*, *Bacteroides*, *Lactobacillus* and *Prevotella* among the three groups. These genera of microbiota are all associated with production of short-chain fatty acids (SCFAs)^3^,^31^. Humans living at high altitude have high energy demands^32^ and pulmonary hypertension^33^. Metabolites produced by microbiota play important roles in host health by being involved in host metabolism^34^. Microbiota can use non-digestible carbohydrates in the colon and produce SCFAs, mainly acetate, propionate and butyrate^34^. SCFAs produced by the gut microbiota not only provide energy, but also decrease blood pressure via olfactory receptor 78 and G protein couple receptor 41^35^. Different genera of microbiota can produce different metabolites. Although propionate exerts a hypotensive effect^34^, the effects of different SCFAs produced by microbiota on blood pressure are still unknown. Our results suggest that gut microbiota potentially influences healthy status by modulating energy harvest and blood pressure response to hypoxia environment at high altitude.

In summary, our study revealed that humans living at high altitude (Tibetans and Chinese Han) had a high abundance of *Firmicutes* and a low abundance of *Bacteroidete*. However, these differences were due to different genus compositions. Compared with the LH group, the changes of the *Firmicutes* and *Bacteroidetes* in the HH group were mainly due to a significant decrease in *Prevotella*. However, in the T group these changes were primarily attributable to significant decreases in *Bacteroides* and *Prevotella* and a significant increase in *Catenibacterium*. Whether these changes of genera could affect adaption to high altitude needs further large-scale studies, taking socioeconomic status, sanitation and exercise intensity into consideration.

## Experimental procedures

### Sampling

We enrolled 12 healthy Tibetan adults (T group) and 12 healthy Han adults (HH group) living in Tibet (Nyingchi, Tibet, with an average altitude of 3100 meters above sea level) and 11 healthy Han adults (LH group) living in Shaanxi (Yangling, Shaanxi, with an average altitude of 450 meters above sea level) for this study. The individuals were all males within a narrow range of age (18–25 years old). They did not have bowel diseases or metabolic diseases and had not taken any antibiotics or probiotics in the three months before the sampling dates. All Tibetan participants were living in Tibet for generations and never left Tibet. Chinese Han in the HH group had lived in Tibet for more than 4 years. Detailed information of each participant is shown in Table 3. The fecal sample was collected from each subject. Fecal samples were frozen immediately after sampling and stored at −80 °C. This study was approved by the Ethics Committee of Agricultural and Animal Husbandry College of Tibet University (Nyingchi, Tibet). Informed consent was provided by all individuals before participation. All experiments were performed in accordance with approved guidelines and regulations.

### DNA extraction

Genomic DNA was extracted from fecal samples by using the E.Z.N.A. stool DNA Kit (Omega, USA) according to the manufacturer’s instructions. The amount of DNA was determined by Nanodrop ND-2000 (Nanodrop, USA). Integrity and size of DNA were checked by 1% (w/v) agarose gel electrophoresis. All DNA samples were stored at −20 °C until further processing.

### Pyrosequencing

The V1-V3 region of the 16S ribosomal RNA (rRNA) gene from each DNA sample was amplified using the bacterial universal forward primer 27F: 5′- AGAGTTTGATCCTGGCTCAG-3′ and the reverse primer 533R: 5′-TTACCGCGGCTGCTGGCAC-3′ containing the A and B sequencing adaptors. The cycling parameters were as follows: 2 min initial denaturation at 95 °C; 25 cycles of denaturation at 95 °C (30 s), annealing at 55 °C (30 s), elongation at 72 °C (30 s); and final extension at 72 °C for 5 min. Three separate PCR reactions of each sample were pooled for pyrosequencing. The PCR products were separated by 2% agarose gel electrophoresis and purified by using the AxyPrepDNA Gel extraction kit (Axygen, USA). Amplicon DNA concentrations were qualified by using a Quant-iT^TM^ PicoGreen double-stranded DNA assay (Invitrogen, Germany). For sequencing, amplicons from each reaction were mixed in equal amounts based on concentrations and subjected to an emulsion PCR as recommended by 454 Life Sciences by using Roche GS FLX Titanium emPCR Kits (Roche, Switzerland). Using the Sequencing Method Manual XLR70 kit (Roche, Switzerland), sequencing was performed on the Roche Genome Sequencer GS-FLX Titanium platform by the Majorbio Bio-Pharm Technology (Shanghai, China).

Valid sequences were sorted into different samples according to barcodes. The programs Seqcln (http://sourceforge.net/projects/seqclean) and Mothur (http://www.mothur.org/wiki/Main_Page) were used for removing low quality sequences (i) that did not perfectly match the PCR primer at the beginning of a read (allowing for 2 errors), (ii) that did not match a barcode and PCR primer at the end of a read (pairwise identity less than 95%), (iii) that were shorter than 200 base pair in length, (iv) that contained ambiguous and homologous nucleotides, or (v) sequence quality score was less than 25 (using minimum average quality score of 25, maximum number of N’s = 0, minimum sequence length = 200).

The high-quality sequences were aligned in accordance with the SILVA alignment (http://www.arb-silva.de/) and similar sequences with a minimum pairwise identity of 97% were clustered into operational taxonomic units (OTUs) using the Mothur program (http://www.mothur.org/wiki/Cluster). OTUs that reached a 97% similarity level were used for diversity (Shannon), richness (Chao), Good’s coverage, and rarefaction curve analyses using the Mothur program. Taxonomical assignments of OTUs exhibiting 97% similarity were performed using the Mothur (http://www.mothur.org/wiki/Classify.seqs) which uses a RDP Naive Bayesian classifier^36^ to calculate the probability of a sequence from a given taxonomy in accordance with SSU rRNA database of SILVA 111 at an 80% confidence level. For each sample, the abundance of bacterial phyla was expressed as the percentage of total sequences. The beta-diversity was performed with QIIME v1.80 by choosing various distance measures^37^. The Adonis test in the R package “vegan” was conducted to test the significance of constrained factors on microbial community^38^. The canonical analysis of principle coordinates (CAP)^39^ was performed by the R package “vegan”. All plots were constructed using the R software. Testing for significantly different phyla and genus among three groups were performed using the Metastats software (http://metastats.cbcb.umd.edu/). The number of permutations used to calculate the *P* value (significance threshold = 0.05, false discovery rate threshold = 0.2) was set to 1000.

### Confirmation of pyrosequencing data by quantitative real-time PCR

In order to further confirm the pyrosequencing data, real-time qPCR reactions were performed as previously described^40^ in a volume of 25 μL containing 10 ng of fecal DNA, but the annealing temperature was changed to 60 °C. A plasmid containing a total bacteria, *Firmicutes* and *Bacteroidetes* 16S rRNA gene from a clone library was diluted from 1 × 10^3^ to 1 × 10^9^ (copies/ml) to construct standard curves for the detection of total bacteria, *Firmicutes* and *Bacteroidetes*. PCR reactions were performed with CFX96 system (Bio-rad, USA) using SYBR Premix Ex TaqII (Takara, Dalian, China). The data are represented as the mean ±SD of triplicate qPCR values.

## Additional Information

**Accession number**: The sequence information in this paper has been deposited in the GenBank Sequence Read Archive with accession number SRP035344.

**How to cite this article**: Li, L. and Zhao, X. Comparative analyses of fecal microbiota in Tibetan and Chinese Han living at low or high altitude by barcoded 454 pyrosequencing. *Sci. Rep*. **5**, 14682; doi: 10.1038/srep14682 (2015).

## Supplementary Material

Supplementary Information

## Figures and Tables

**Table 1:** Summary of pyrosequencing data.

Group	Reads	OTUs	Chao	Coverage	Shannon
LH	7066 ± 1086	1597 ± 152	4159 ± 568	0.85 ± 0.02	5.56 ± 0.44
HH	6225 ± 827	1450 ± 232	3670 ± 620	0.85 ± 0.03	5.67 ± 0.40
T	7492 ± 1232	1732 ± 252	4521 ± 758	0.84 ± 0.03	5.54 ± 0.50

The number of reads, OTUs, richness estimator Chao and Ace, diversity estimator Shannon were calculated at the 97% similarity level. LH = Chinese Han living in Shaanxi; HH = Chinese Han living in Tibet; HH = Tibetans.

**Table 2:** Relative abundance at the phylum level and statistical significance among three groups.

Phylum	Relative abundance (%)			Significance		
	LH	HH	T	LHvsHH	LHvsT	HHvsT
*Firmicutes*	75.311	90.453	92.770	*	**	NS
*Bacteroidetes*	23.591	7.983	4.690	*	**	NS
*Actinobacteria*	0.802	0.210	0.616	*	NS	NS
*Cyanobacteria*	0.008	0.001	0.015	NS	NS	*
*Fusobacteria*	0.004	0.020	0.001	NS	NS	*
*Verrucomicrobia*	0.003	0.004	0.012	NS	*	NS

LH = Chinese Han living in Shaanxi; HH = Chinese Han living in Tibet; T = Tibetans.

Significance: NS > 0.05, *≤0.05, **≤0.01.

**Table 3:** Basic information of experimental subjects.

Sample ID^1^	Nationality	Age	Living in Tibet	BMI^2^
HH1	Han	23	51 months	22
HH2	Han	22	51 months	19.3
HH3	Han	22	51 months	18.1
HH4	Han	23	51 months	21.5
HH5	Han	24	51 months	17.6
HH6	Han	23	51 months	20.9
HH7	Han	25	51 months	21.2
HH8	Han	22	51 months	18.7
HH9	Han	24	72 months	24.3
HH10	Han	21	60 months	18.7
HH11	Han	23	51 months	19.7
HH12	Han	22	49 months	20.3
T1	Tibetan	22	Always	17.8
T2	Tibetan	21	Always	18.2
T3	Tibetan	20	Always	18.6
T4	Tibetan	21	Always	19.5
T5	Tibetan	18	Always	22.2
T6	Tibetan	25	Always	23.1
T7	Tibetan	20	Always	22
T8	Tibetan	20	Always	20.8
T9	Tibetan	20	Always	20.3
T10	Tibetan	21	Always	20.2
T11	Tibetan	20	Always	21.5
T12	Tibetan	20	Always	19.4
LH1	Han	20	Never	25.1
LH2	Han	19	Never	24.9
LH3	Han	21	Never	22.7
LH4	Han	20	Never	21.9
LH5	Han	20	Never	18.4
LH6	Han	19	Never	18.6
LH7	Han	19	Never	21.3
LH8	Han	20	Never	18.2
LH9	Han	22	Never	24.2
LH10	Han	22	Never	23.6
LH12	Han	25	Never	22.3

^1^LH = Chinese Han living in Shaanxi; HH = Chinese Han living in Tibet; HH = Tibetans.

^2^BMI = body mass index.

## References

[b1] Ley R. E., Peterson D. A. & Gordon J. I. Ecological and evolutionary forces shaping microbial diversity in the human intestine. Cell 124, 837–848 (2006). 16497592 10.1016/j.cell.2006.02.017

[b2] Sekirov I., Russell S. L., Antunes L. C. & Finlay B. B. Gut microbiota in health and disease. Physiol Rev 90, 859–904 (2010). 20664075 10.1152/physrev.00045.2009

[b3] De Filippo C. . Impact of diet in shaping gut microbiota revealed by a comparative study in children from Europe and rural Africa. Proc Natl Acad Sci USA 107, 14691–14696 (2010). 10.1073/pnas.1005963107 PMC2930426 20679230

[b4] Lin A. . Distinct distal gut microbiome diversity and composition in healthy children from Bangladesh and the United States. PloS ONE 8, e53838 (2013). 23349750 10.1371/journal.pone.0053838 PMC3551965

[b5] Yatsunenko T. . Human gut microbiome viewed across age and geography. Nature 486, 222–227 (2012). 22699611 10.1038/nature11053 PMC3376388

[b6] Arumugam M. . Enterotypes of the human gut microbiome. Nature 473, 174–180 (2011). 21508958 10.1038/nature09944 PMC3728647

[b7] Chatterjee B. & Thakur S. S. Microbial profiling, extend ethnicity of human microbiome. Nature 487, 39–39 (2012). 22763544 10.1038/487039d

[b8] Kwok L. Y. . Characterization of fecal microbiota across seven chinese ethnic groups by quantitative polymerase chain reaction. PLoS ONE 9, e93631 (2014). 24699404 10.1371/journal.pone.0093631 PMC3974763

[b9] Khachatryan Z. A. . Predominant role of host genetics in controlling the composition of gut microbiota. PLoS ONE 3, e3064 (2008). 18725973 10.1371/journal.pone.0003064 PMC2516932

[b10] Goodrich J. K. . Human genetics shape the gut microbiome. Cell 159, 789–799 (2014). 25417156 10.1016/j.cell.2014.09.053 PMC4255478

[b11] Kleessen B. . Microbial and immunological responses relative to high-altitude exposure in mountaineers. Med Sci Sports Exerc 37, 1313–1318 (2005). 16118577 10.1249/01.mss.0000174888.22930.e0

[b12] Adak A., Maity C., Ghosh K., Pati B. R. & Mondal K. C. Dynamics of predominant microbiota in the human gastrointestinal tract and change in luminal enzymes and immunoglobulin profile during high-altitude adaptation. Folia Microbiol 58, 523–528 (2013). 23536261 10.1007/s12223-013-0241-y

[b13] Wu T. Y. & Kayser B. High altitude adaptation in Tibetans. High Alt Med Biol 7, 193–208 (2006). 16978132 10.1089/ham.2006.7.193

[b14] Beall C. M. . Natural selection on EPAS1 (HIF2α) associated with low hemoglobin concentration in Tibetan highlanders. Proc Natl Acad Sci USA 107, 11459–11464 (2010). 20534544 10.1073/pnas.1002443107 PMC2895075

[b15] Yi X. . Sequencing of 50 human exomes reveals adaptation to high altitude. Science 329, 75–78 (2010). 20595611 10.1126/science.1190371 PMC3711608

[b16] Peng Y. . Genetic variations in Tibetan populations and high-altitude adaptation at the Himalayas. Mol biol Evol 28, 1075–1081 (2011). 21030426 10.1093/molbev/msq290

[b17] Lorenzo F. R. . A genetic mechanism for Tibetan high-altitude adaptation. Nat Genet. 46, 951–956 (2014). 25129147 10.1038/ng.3067 PMC4473257

[b18] Ge K., Zhai F. & Wang Q. Effect of nationality on dietary pattern and meal behavior in China. Am J Clin Nutr 65, 1290S–1294S (1997). 9094935 10.1093/ajcn/65.4.1290S

[b19] Tashi N., Yawei T. & Zeng X. Q. Food Preparation from hulless barley in Tibet. Advance in Barley Sciences. Springer: Netherlands, , pp. 151–158 (2013).

[b20] Wu T. Life on the high Tibetan plateau. High Alt Med Biol 5, 1–2 (2004). 15072710 10.1089/152702904322963609

[b21] Muza S. R., Beidleman B. A. & Fulco C. S. Altitude preexposure recommendations for inducing acclimatization. High Alt Med Biol 11, 87–92 (2010). 20586592 10.1089/ham.2010.1006

[b22] Peng Q. Q. . Physiological responses and evaluation of effects of BMI, smoking and drinking in high altitude acclimatization: a cohort study in Chinese Han young males. PLoS ONE 8, e79346 (2013). 24260204 10.1371/journal.pone.0079346 PMC3832642

[b23] White J. R., Nagarajan N. & Pop M. Statistical Methods for Detecting Differentially Abundant Features in Clinical Metagenomic Samples. PLoS Comput Biol 5, e1000352 (2009). 19360128 10.1371/journal.pcbi.1000352 PMC2661018

[b24] Ley R. E., Turnbaugh P. J., Klein S. & Gordon J. I. Microbial ecology: human gut microbes associated with obesity. Nature 444, 1022–1023 (2006). 17183309 10.1038/4441022a

[b25] WHO expert consultation. Appropriate body-mass index for Asian populations and its implications for policy and intervention strategies. Lancet 363, 157–163 (2004). 14726171 10.1016/S0140-6736(03)15268-3

[b26] Murphy E. F. . Composition and energy harvesting capacity of the gut microbiota: relationship to diet, obesity and time in mouse models. Gut 59, 1635–1642 (2010). 20926643 10.1136/gut.2010.215665

[b27] Furet J. P. . Differential adaptation of human gut microbiota to bariatric surgery-induced weight loss: links with metabolic and low-grade inflammation markers. Diabetes 59, 3049–3057 (2010). 20876719 10.2337/db10-0253 PMC2992765

[b28] Paillard D. . Relation between phylogenetic position, lipid metabolism and butyrate production by different Butyrivibrio-like bacteria from the rumen. Antonie Van Leeuwenhoek 91, 417–422 (2007). 17077990 10.1007/s10482-006-9121-7

[b29] Konopka A. What is microbial community ecology? ISME J 3, 1223–1230 (2009). 19657372 10.1038/ismej.2009.88

[b30] Wu G. D. . Linking long-term dietary patterns with gut microbial enterotypes. Science 334, 105–108 (2011). 21885731 10.1126/science.1208344 PMC3368382

[b31] Ramakrishna B. S. Role of the gut microbiota in human nutrition and metabolism. J Gastroenterol Hepatol 28, 9–17 (2013). 24251697 10.1111/jgh.12294

[b32] Kayser B. & Verges S. Hypoxia, energy balance and obesity: from pathophysiological mechanisms to new treatment strategies. Obes Rev 14, 579–592 (2013). 23551535 10.1111/obr.12034

[b33] Bailey D. M. . High-altitude pulmonary hypertension is associated with a free radical-mediated reduction in pulmonary nitric oxide bioavailability. J Physiol 1588, 4837–4847 (2010). 20876202 10.1113/jphysiol.2010.194704 PMC3010150

[b34] Tremaroli V. & Bäckhed F. Functional interactions between the gut microbiota and host metabolism. Nature 489, 242–249 (2012). 22972297 10.1038/nature11552

[b35] Pluznick J. L. . Olfactory receptor responding to gut microbiota-derived signals plays a role in renin secretion and blood pressure regulation. Proc Natl Acad Sci USA 110, 4410–4415 (2013). 23401498 10.1073/pnas.1215927110 PMC3600440

[b36] Wang Q., Garrity G. M., Tiedje J. M. & Cole J. R. Naive Bayesian classifier for rapid assignment of rRNA sequences into the new bacterial taxonomy. Appl Environ Microbiol 73, 5261–5267 (2007). 17586664 10.1128/AEM.00062-07 PMC1950982

[b37] Caporaso J. G. . QIIME allows analysis of high-throughput community sequencing data. Nature Methods 7, 335–336 (2010). 20383131 10.1038/nmeth.f.303 PMC3156573

[b38] Oksanen J. . vegan: Community Ecology Package (Comprehensive R Archive Network). R package Version 1, 17–6 (2011).

[b39] Anderson M. J. & Willis T. J. Canonical analysis of principle coordinates: a useful method of constrained ordination for ecology. Ecology 84, 511–525 (2003).

[b40] Bacchetti De Gregoris T., Aldred N., Clare A. S. & Burgess J. G. Improvement of phylum-and class-specific primers for real-time PCR quantification of bacterial taxa. J Microbiol Methods 86, 351–356 (2011). 21704084 10.1016/j.mimet.2011.06.010

