# Supplementary material for: Comparative analyses of fecal microbiota in Tibetan and Chinese Han living at low or high altitude by barcoded 454 pyrosequencing
Source: Sci Rep. 2015 Oct 7;5:14682. doi: 10.1038/srep14682 (PMC4595765; doi:10.1038/srep14682)
Supplement: Supplementary Information [file srep14682-s1.pdf]

Title: Comparative analyses of fecal microbiota in Tibetan and Chinese Han living in low or high altitude by barcoded 454 pyrosequencing

Authors: Long Li[1,2], Xin Zhao[1,3,*]

Address: [1]College of Animal Science and Technology, Northwest A&F University, Yangling, Shaanxi, People's Republic of China

[2]College of Animal Science, Agricultural and Animal Husbandry College of Tibet University, Nyingchi, Tibet, People's Republic of China

[3]Department of Animal Science, McGill University, 21,111 Lakeshore, Ste. Anne de Bellevue, Quebec, Canada, H9X 3V9

Table S1. Pyrosequencing data and estimator index of experimental subjects

Sample ID	Reads	OTUs	Chao	Coverage	Shannon
HH1	5542	1441	3744	0.831108	6.01
HH2	7594	1593	4314	0.858836	5.47
HH3	5538	1438	3455	0.831347	5.89
HH4	6797	1419	3570	0.863469	5.37
HH5	6651	1691	4250	0.837167	6.14
HH6	4663	837	2172	0.884195	4.64
HH7	6745	1527	4270	0.84937	5.56
HH8	5563	1318	3205	0.846486	5.63
HH9	6202	1562	4011	0.835214	5.95
HH10	6872	1600	3508	0.856665	5.98
HH11	5707	1292	3558	0.852111	5.72
HH12	6823	1676	4342	0.837608	5.73
LH1	9487	1697	4525	0.87973	4.57
LH2	6938	1717	4361	0.836552	5.92
LH3	5838	1460	4180	0.832306	5.74
LH4	8128	1677	4430	0.865157	5.57
LH5	7241	1720	4288	0.841873	5.67
LH6	6068	1578	4153	0.822512	5.7
LH7	7175	1762	4506	0.838328	5.77
LH8	5635	1465	4088	0.826974	5.95
LH9	5983	1627	4499	0.815477	5.84
LH10	8206	1250	2512	0.909944	4.86
LH12	7032	1611	4207	0.84727	5.58
T1	8066	1886	4829	0.846268	6.08
T2	5279	1164	3002	0.850161	4.88
T3	7436	1670	4332	0.852743	5.7
T4	9617	1588	4166	0.892794	5.46
T5	6867	1440	3286	0.865152	5.36
T6	8680	1704	4640	0.866935	4.91
T7	9153	1998	5458	0.852835	5.06
T8	7460	1950	5434	0.822654	5.74
T9	7117	1642	4487	0.844878	4.98
T10	6173	1817	4680	0.803013	6.11
T11	7236	1994	4766	0.819928	6.13
T12	6825	1936	5174	0.810549	6.11

The number of reads, OTUs, richness estimator Chao, and diversity estimator Shannon were calculated at the 97% similarity level. LH = Chinese Han living in Shaanxi; HH = Chinese Han living in Tibet; T = Tibetan.

Table S2. The P-values of ethnic and altitude on microbiota composition with various distance matrixes

Factors	P-values					
	Weighted UniFrac	Uneighted UniFrac	Abundance_ Jaccard	Binary_ Jaccard	Bray_ curtis	Hellinger
Ethnic	0.0089	0.0001	0.0001	0.0003	0.0011	0.0003
Altitude	0.0028	0.0091	0.0268	0.126	0.1328	0.708

Table S3. Intake frequencies of selected main foods (times per week)

Group[1] \ Food	Cereals	Tubers	Legumes	Meat	Dairy	Vegetables
LH (n=11)	15.2±3.9	9.2±3.2	4.4±1.2	14.0±1.9[b]	6.3±4.3[b]	14.8±7.3[a]
HH (n=12)	16.9±5.8	10.6±2.2	4.3±2.1	13.9±2.5[b]	7.8±5.1[b]	13.1±6.5[a]
T (n=12)	15.3±5.4	9.4±5.3	3.3±1.4	16.8±3.0[a]	11.9±5.3[a]	9.2±3.1[b]

[1]LH = Chinese Han living in Shaanxi; HH = Chinese Han living in Tibet; T = Tibetans.

[a,b] Values with different superscripts within the same column are significantly different ($p < 0.05$).

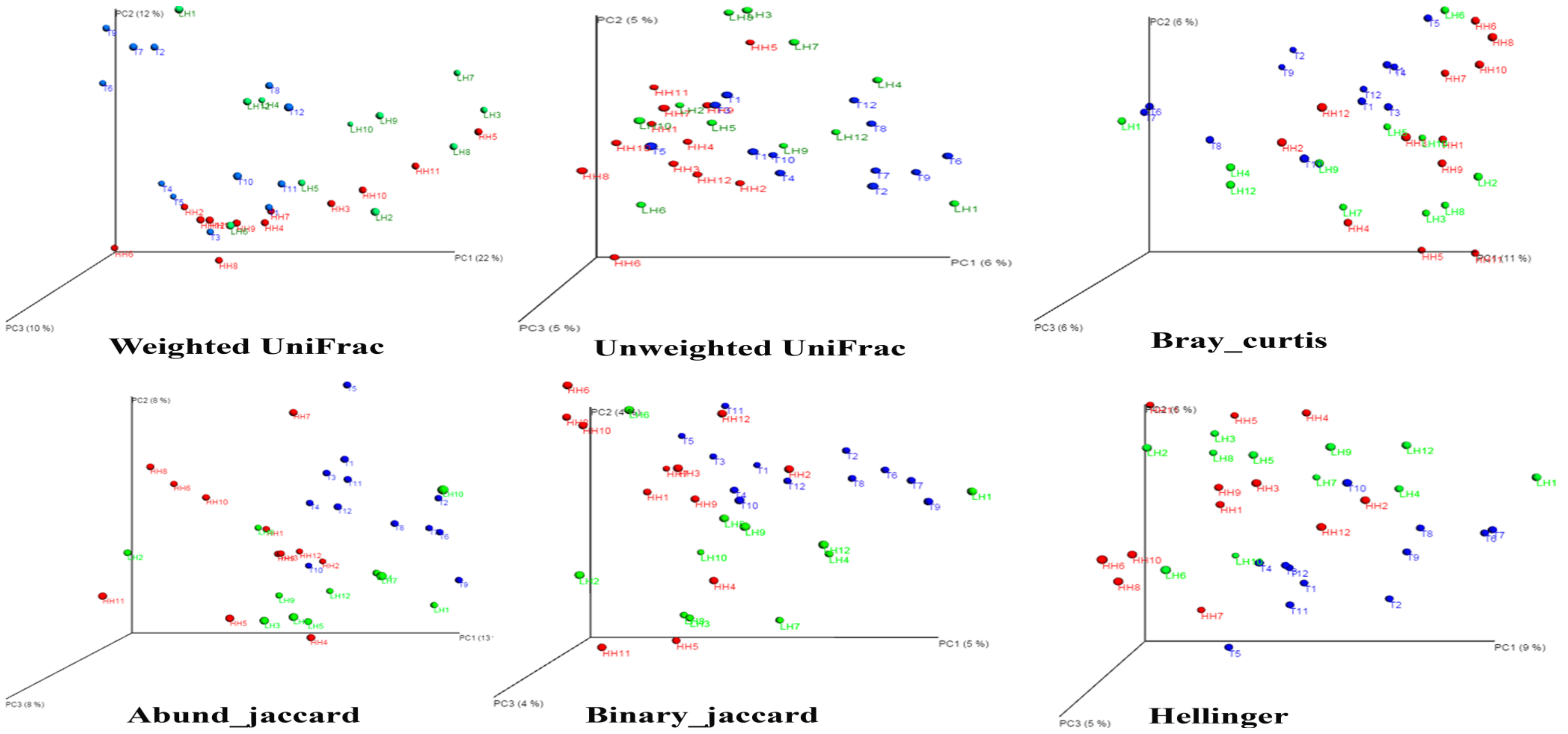

Figure S1. Beta-diversity analysis of all samples by unconstrained ordination methods with various distance matrixes. LH = Chinese Han living in Shaanxi (blue dots); HH = Chinese Han living in Tibet (green dots); T = Tibetans (blue dots).
